# Soil nitrogen-related functional genes undergo abundance changes during vegetation degradation in a Qinghai-Tibet Plateau wet meadow

**DOI:** 10.1128/aem.00813-24

**Published:** 2024-09-20

**Authors:** Jianan Du, Weiwei Ma, Guang Li, Wenhua Chang, Longyong Chun

**Affiliations:** 1 College of Forestry, Gansu Agricultural University, Lanzhou, China; Colorado School of Mines, Golden, Colorado, USA

**Keywords:** wetland ecosystem, nitrogen functional genes, vegetation degradation process, nitrogen cycling

## Abstract

Climate change and anthropogenic activities have significantly contributed to the degradation of wet meadows on the Qinghai-Tibet Plateau (QTP). Soil nitrogen (N) availability is a crucial determinant of the productivity of wet meadow vegetation. Furthermore, soil microbial nitrogen functional genes (NFGs) are critical in the transformation of soil N. Nevertheless, the dynamics of NFGs in response to vegetation degradation, as well as the underlying drivers, remain poorly understood. In this study, wet meadows at varying levels of vegetation degradation on the QTP, categorized as non-degraded (ND), slightly degraded (SD), moderately degraded (MD), and heavily degraded (HD), were examined. Soil samples from depths of 0 to 10 cm and 10 to 20 cm were collected during different growth cycles (June 2020, August 2020, and May 2021). The analysis focused on NFGs involved in organic nitrogen fixation (*nifH*), archaeal and bacterial ammonia oxidation (*amoA-AOA* and *amoA-AOB*, respectively), and nitrite reduction (*nirK*), utilizing real-time fluorescence quantitative PCR. Our findings indicate a significant decline in the abundance of NFGs with intensified vegetation degradation, exhibiting notable spatial and temporal fluctuations. Specifically, the relative NFGs followed the pattern: *nirK* > *amoA-*AOA > *amoA-*AOB > *nifH*. Redundancy analysis revealed that vegetation cover was the primary regulator of NFGs abundance, accounting for 56.1%–57% of the variation. Additionally, soil total nitrogen, pH, and total phosphorus content were responsible for 38.5%, 28.2%, and 7% of the variability in NFGs, respectively. The (*amoA-AOA + amoA-AOB + nirK*) ratios associated with effective N transformation indicated that the vegetation degradation process moderately increased the nitrification potential.

**IMPORTANCE:**

Our research investigates how the degradation of meadows affects the tiny organisms in soil that help plants use nitrogen, which is essential for their growth. In the Qinghai-Tibet Plateau, a region known for its unique ecosystems, we found that as meadows deteriorate—due to climate change and human activities—the number of these beneficial organisms significantly decreases. This decline could reduce soil fertility, impacting plant life and the overall health of the ecosystem. Understanding these changes helps us grasp how environmental pressures influence soil and plant health. Such knowledge is crucial for developing strategies to preserve these vulnerable ecosystems and ensure they continue to sustain biodiversity and provide resources for local communities.

## INTRODUCTION

Wetlands, despite covering a mere fraction of the Earth’s surface, are acknowledged as highly efficient ecosystems that are vital for global nutrient cycling (1, 2). Central to this efficiency is the vegetation, which drives the material cycle and exchange of energy in wetland ecosystems. The health and progression of vegetation directly influence the stability and evolutionary trajectory of wetland ecosystems. However, due to global warming and intensified human activities, wetlands worldwide are undergoing significant vegetation degradation or even experiencing loss (3–5). This degradation impacts the potential for nitrogen (N) turnover in these areas. It is important to note that N turnover potential can vary during different stages of vegetation degradation. Thus, to accurately gauge the productivity and quality of wetland ecosystems, it is imperative to understand how N turnover potential responds to vegetation degradation.

In wetland ecosystems, soil N turnover potential is primarily driven by N-cycling microbe (6). This process involves four principal components: N fixation in the soil (conversion of atmospheric N to organic N), ammonification [transformation of organic N to ammonium nitrogen (NH_4_^+^-N)], and both nitrification and denitrification [interconversion among NH_4_^+^-N, nitrate nitrogen (NO_3_^−^-N), and nitrite forms] (6). Consequently, the response of key attributes specifically gene abundance and relative gene abundance of N-cycling microbial communities to various environmental factors, vegetation conditions, and ecosystems can be leveraged to quantitatively analyze the N-cycling process. Specifically, variations in the abundance of nitrogen-functioning genes (NFGs), as influenced by the N cycle, provide valuable insights into the dynamic state of N-cycling (7–9). As a critical marker of the N fixation process, the abundance of the *nifH* gene is commonly employed to assess soil N fixation capacity. The gene abundances of ammonia-oxidizing microbial communities, specifically *amoA-AOB* and *amoA-AOA*, are indicators of the soil nitrification process, where ammonia is converted into nitrate. Additionally, the abundance of nitrite reductase genes (*nirK*/*nirS*) is often used to assess the denitrification process (8, 10).

Vegetation acts as a direct utilizer and regulator of soil nutrients. The growth process of vegetation alters the soil structure and nutrient content, directly influencing soil microbial activity, which In turn affects the dynamic equilibrium of soil nutrients (6, 11, 12). Some studies indicate that vegetation degradation in alpine wetlands, such as biomass reduction and species turnover processes, greatly affects nutrient turnover in alpine wetland soils (13, 14). These alterations impact oxygen transport efficiency and nutrient input. Concurrently, soil chemical properties also shift across vegetation degradation gradients (15, 16). The process of vegetation degradation alters the soil structure and nutrient content, leading to a gradual decline in overall soil physicochemical properties. This decline subsequently affects the abundance of NFGs and disrupts or alters the N-cycling process. Reports of available studies showed that degradation in wetlands can lead to a decreased abundance of denitrifying microorganisms (like *nirS* genes) (17). However, the impact of vegetation degradation in alpine wetlands on the various functions of NFGs remains unclear. There is an insufficient understanding of whether specific strategies exist within NFGs to respond to vegetation degradation.

Nearly one-third of China’s wetlands are situated on the Qinghai-Tibet Plateau (QTP). Spanning 2.5 million square kilometers, wet meadows form a significant part of the QTP wetlands (16, 18, 19). This area is a key ecological zone for maintaining the dynamic balance of carbon (C) and N pools, as well as for soil and water conservation in the alpine region (20). However, these wet meadow areas have undergone degradation due to climate change, drastic changes in local hydrological conditions, and irrational human activities (21, 22), leading to losses in C and N as well as a decline in biodiversity. Additionally, since the 1970s, there has been a noticeable decrease in the water table (23) and an increase in vegetation degradation, exacerbated by the drainage of original wetlands and increased grazing (24). Furthermore, over time, the vegetation species in the QTP wet meadows have shifted from hydrophytes to mesophytes and xerophytes, indicating significant degradation (21). As a result, the loss and degradation of the initially dominant species, coupled with the further deterioration of hydrological conditions, have led to a marked decline in the vegetation quality of the original wet meadows, resulting in a decreased overall ecological function of wet meadows (15, 16).

While previous studies have utilized soil enzymes like amylase and peroxidase to monitor vegetation impacts (21), our study leverages the precision of genetic markers (NFGs) to assess how these impacts at the molecular level correlate with changes in N cycling. This approach offers a more direct measure of microbial responses to environmental stressors, which, according to our current data, has prompted the emission of greenhouse gases and reduced the activity of soil amylase and peroxidase enzymes (16, 25). This reduction in enzyme activity intensifies the potential for global warming (16). We specifically target NFGs in our study to uncover the detailed molecular mechanisms through which N cycling processes adapt to environmental changes, such as alterations in vegetation cover and soil structure (26). However, the specific interactions and relationships between these NFGs and the degradation of alpine wetland vegetation remain underexplored. Therefore, it is imperative to conduct more in-depth research to determine the effectiveness of N utilization in the context of vegetation degradation. Consequently, the N cycling processes involving NFGs may adapt in response to environmental changes triggered by alterations in vegetation and soil structure. However, the temporal dynamics of these microorganisms in relation to vegetation degradation in alpine wetlands remain underexplored. Therefore, gaining a deeper understanding of N availability and storage potential across different stages of vegetation degradation and at various time points is of paramount importance. In this study, we employed an experimental area previously established on the QTP, which included four levels of vegetation degradation [non-degraded (ND), slightly degraded (SD), moderately degraded (MD), and heavily degraded (HD)] in wet meadows (16). Our objective was to investigate the impact of vegetation degradation in alpine wetlands on the variations in the abundance of NFGs in order to ascertain how the processes of wetland degradation influence soil N-cycling dynamics. We proposed the following hypotheses: (i) NFGs abundance would decrease in response to vegetation degradation and the consequent reduction in soil organic matter input. (ii) The abundance of NFGs exhibited substantial variation in response to soil water and temperature regulation, peaking during favorable conditions (coinciding with peak vegetation growth periods) and decreasing as water and temperature conditions became less favorable (during the pre-vegetation growth period). Furthermore, we hypothesized that fluctuations in the abundance of NFGs would be significantly influenced by changes in the content of C and N fractions in the soil.

## MATERIALS AND METHODS

### Plot selection and design

The present study was conducted in an experimental area established in May 2016 at the Gahai-Zecha International Nature Reserve, located in Gansu Province, People’s Republic of China (34°16'N, 102°26'E) (21). The Gahai-Zecha International Nature Reserve, listed as one of the internationally important protected wetlands (27), is situated on the northeastern edge of the QTP. This location is particularly sensitive to N cycling due to its prolonged exposure to low temperatures and nitrogen-deficient conditions (28). These environmental stresses are compounded by human disturbances, including climate change and excessive grazing, which have surpassed the ecosystem’s theoretical grazing capacity and led to significant vegetation degradation (24). The degradation is most evident in the sparse surface vegetation characteristic of the wet meadows, making it an ideal microcosm for studying similar challenges faced by wetlands globally (29, 30). This setting provides a pertinent case study for understanding the broader ecological impacts and necessary adaptive restoration strategies. Our prior studies have confirmed that this degradation not only diminishes the total organic carbon and its components in these alpine wet meadows but also degrades their carbon sequestration ability and soil quality, thereby exacerbating pressure on the atmospheric environment (15, 21, 25).

We centered our study around Gahai-Lake and extended four sample strips outward, each representing different levels of vegetation degradation: ND, SD, MD, and HD (Fig. S1). Detailed characteristics of these plots are provided in Table S1. The experimental site is characterized by a cold temperate continental climate, with an annual average temperature of 2.9°C and annual precipitation totaling 593 mm (16). More details about the experimental area are described in our previous study and will not be repeated here (15, 16, 21, 25).

### Sample collection

On 20 June 2020, during the rapid growth stage of plants (June to July), 20 August 2020, when vegetation reached maturity, and 18 May 2021, as vegetation began its growth phase (May to June), field soil sampling was conducted to analyze the abundances of NFGs. We established three replicate plots for each level of degradation, configuring each plot as a 5 m × 5 m square and maintaining a minimum distance of 5 m between replicates to ensure representativeness. In total, 12 study plots (4 levels of degradation × 3 replicates) were set up. After establishing the sample plots, we employed a diagonal sampling method involving two points at the diagonal of each plot, from which we randomly selected three points along the diagonal for sampling. Once the soil sampling locations were determined, we manually cleared dead material and native vegetation from the surface. We then used a soil auger with a radius of 2 cm to collect soil samples in sections from the 0 to 10 cm and 10 to 20 cm soil layers. After removing stones and vegetative roots from the soil samples collected at the same points, we combined the samples from the same soil horizons into a composite sample. This composite sample was then equally divided into two parts. One portion was placed in a bio-ice pack and taken to the laboratory for soil physicochemical analysis. The other portion of the soil sample was quickly transferred into pre-sterilized, tinfoil-wrapped centrifuge tubes and then stored in −80°C liquid N in an ultra-low-temperature freezer for later soil microbial analysis (DNA extraction). Additionally, we randomly selected five smaller replicate plots measuring 50 cm × 50 cm within each sample plot to assess vegetation cover, composition, and both aboveground biomass (AB) and belowground biomass (BB). Aboveground biomass is determined by uniformly collecting vegetation from a designated small sample plot, drying it, and then weighing it. Belowground biomass is assessed by extracting inter-root samples from the same soil depth using a root auger with a radius of 5 cm. The collected inter-roots are then cleaned, dried in an oven at 60°C for 36 hours, and weighed to determine the belowground biomass. Detailed information on the different vegetation degradation plots is given in Table 1.

**TABLE 1:** Changes in plant properties during the levels of degraded vegetation^*a*^

Vegetation degradation level	Coverage (%)	Height (cm)	Aboveground biomass (g m^−2^)	Belowground biomass (g m^−2^)	
ND	95.33 ± 3.05a	16.71 ± 2.98a	625.41 ± 2.17a	0–10 cm	2504.07 ± 21.60a
				10–20 cm	968.73 ± 103.72a
SD	80.66 ± 5.03b	13.02 ± 2.24b	444.39 ± 12.76b	0–10 cm	1074.26 ± 234.70b
				10–20 cm	717.47 ± 179.76b
MD	43.67 ± 9.07c	7.43 ± 0.97c	376.00 ± 29.53c	0–10 cm	700.99 ± 142.38c
				10–20 cm	506.48 ± 26.64b
HD	–	–	–	0–10 cm	–
				10–20 cm	–

^*a*^ Values are expressed as mean ± SD. Coverage, height, and aboveground under different lowercase letters under the same degradation degree exists significant difference (*P* < 0.05). Different lowercase letters in belowground biomass indicated that there were significant differences among the same soil layers under different degradation degrees (*P* < 0.05). A dash indicates that the surveyed data are close to zero.

### Soil physicochemical parameters and nitrogen component analysis

**Fig 1:**
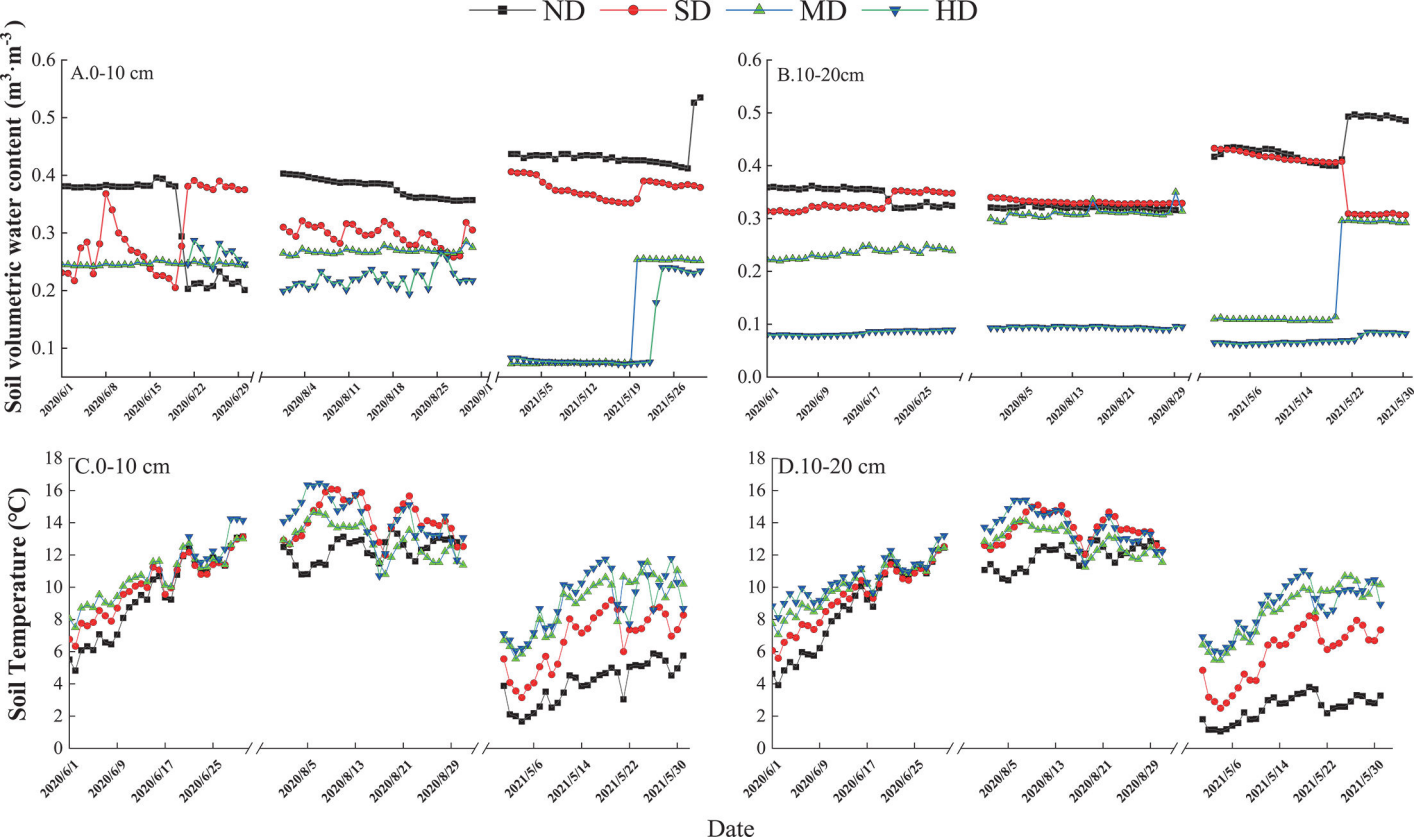
Daily changes in soil volume moisture content (A and B) and soil temperature (C and D) during the measurement period of 2020 and 2021.

To accurately monitor volumetric soil water content and soil temperature, we employed Em50 G data loggers from Decagon, America. Moisture and temperature probes were installed in both the 0–10 cm and 10–20 cm soil layers for all three replicates within each specific level of vegetation degradation. This setup ensured detailed and consistent data collection across different soil depths and degradation levels. This setup was used to monitor changes in soil volumetric water content and temperature, with data recorded every 10 minutes. The results of these measurements are displayed in Fig. 1A through C. The soil physicochemical parameters assessed in this study primarily included pH, soil organic carbon (SOC), total phosphorus (TP), and total potassium (TK), which were used to evaluate the baseline nutrient status of the sample sites. Additionally, various soil N fractions were determined, including total nitrogen (TN), NH_4_^+^-N and NO_3_^−^-N, to provide a comprehensive analysis of the soil N profile. The determination of the above indicators was carried out with reference to previous studies (31, 32). Bulk density (BD) was measured with the core method, which consists of inserting a tilted stainless steel ring into the soil to collect a known volume of soil, which is then dried and weighed (21). Analysis of soil physicochemical parameters was conducted exclusively on 20 August 2020, while concentrations of soil N components were analyzed during all sampling periods. Details of these soil physicochemical parameters are presented in Table S2.

### DNA extraction and quantification of functional genes by real-time PCR

DNA was extracted from 0.25 to 0.5 g of fresh sediment using the E.Z.N.A. Soil DNA Kit (Omega Biotek, Norcross, GA, USA) in accordance with the manufacturer’s instructions. The integrity of the extracted DNA was verified through 1% agarose gel electrophoresis. Following verification, the DNA was stored at −20°C until further analysis (7).

The abundances of NFGs, specifically *nifH*, *amoA-AOB*, *amoA-AOA*, and *nirK*, were quantified using the CFX96 Real-Time PCR Detection System (Bio-Rad, USA). Standard curves for each target gene were established through real-time PCR, employing a 10-fold serial dilution of plasmid DNA containing the respective gene. Each 15 µL reaction mixture consisted of 7.5 µL of SYBR Green qPCR Master Mix (Jizhen Biosystems, China), 0.7 µL of each primer (forward and reverse) at a 20 µM concentration, 1 µL of the DNA template, and 5.1 µL of sterile water. The reaction efficiencies for the quantitative real-time PCR (qPCR) ranged from 79% to 93%, and the *R*^2^ values for the standard curves varied from 0.990 to 0.999. Detailed primer information for the target genes is provided in Table S3. The abundance of the NFGs was expressed as copies per gram of dry soil. All samples were analyzed in triplicate to ensure the accuracy and reproducibility of the results.

### Data analysis

In this study, the aggregate abundance of total nitrogen functional genes (TNFGs) was quantified by aggregating the abundances of individual N-cycling genes. The proportional abundance of each N cycle gene was ascertained by calculating the ratio of its abundance to the TNFGs, providing insight into the specific contributions of distinct N cycle gene populations to N-cycling dynamics. To examine variations in soil physicochemical attributes, vegetation characteristics, and NFGs abundance across different sampling times and plots, both analysis of variance (ANOVA) and least significant difference tests were utilized. Repeated measures ANOVA, incorporating sampling time as the repeated measure, was applied to evaluate fluctuations in NFG abundance along vegetation degradation gradients. Statistically, differences were deemed highly significant when *P* < 0.01 and significant when *P* < 0.05. Furthermore, redundancy analysis (RDA) was executed to elucidate the correlations between soil physicochemical properties, vegetation characteristics, and NFGs. All statistical analyses were conducted using SPSS version 24.0, and redundancy analysis was performed with CANOCO 5.0. The results were presented as mean ± SE, derived from three replicates.

## RESULTS

### Environmental factors

#### Plant and soil properties

Vegetation degradation adversely affected plant coverage and biomass, leading to reduced coverage and both above- and belowground biomasses in degraded plots compared to ND plots (Table 1). Plant coverage and aboveground biomass significantly decreased with increasing levels of degradation, reaching their lowest at MD plots, with values of 43.67% and 376.00 g m^−2^, respectively. Belowground biomass diminished with increasing levels of degradation, with the highest values recorded in the ND plots. Specifically, the biomass in the 0–10 cm layer ranged from 2504.07 g m^−2^ to 968.73 g m^−2^, while in the 10–20 cm layer, it ranged from 700.99 g m^−2^ to 506.48 g m^−2^ (Table 1). In the same soil layers, TP and SOC contents were significantly higher in the ND and SD samples compared to MD and HD samples (Table S2). TK content exhibited greater stability across levels of degradation. The pH value fluctuated in a small range, ranging from 7.94 to 7.65 and 7.73 to 7.95 in 0–10 cm and 10–20 cm soil layers, respectively. BD increased with both the level of degradation and soil depth.

#### Soil nitrogen components

**Fig 2:**
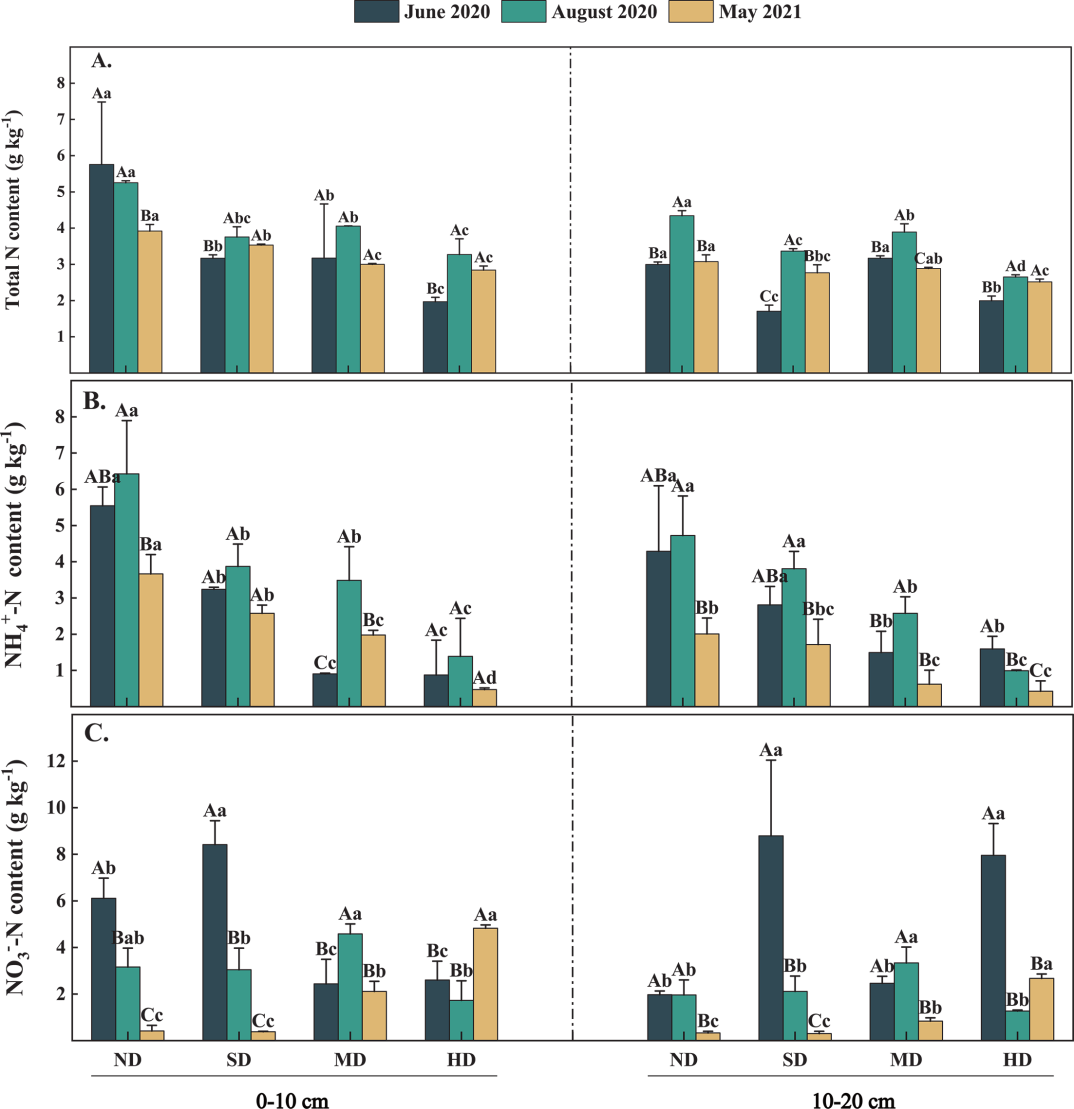
Dynamic changes of soil nitrogen components in four wetland degradation types during the observation period of 2020–2021. On the bar chart, different lowercase letters indicate that there are significant differences between different points at the same time, and different uppercase letters indicate that there are significant differences between the same points at different times, the significance is *P* < 0.05, and vertical bar is SE (*n* = 3).

Variations in TN, NH_4_^+^-N, and NO_3_^−^-N contents exhibited significant fluctuations across different sampling times and levels of vegetation degradation. Specifically, TN content in the 0–10 cm soil layer at the ND plot (3.92–5.79 g kg^−1^) was significantly higher than those at the SD (3.17–3.75 g kg^−1^), MD (3.00–4.05 g kg^−1^), and HD (1.97–3.27 g kg^−1^) plots (*P* < 0.05). In the 10–20 cm layer, TN content at the ND plot (2.99–4.34 g kg^−1^) was also significantly higher compared to the SD (1.71–3.36 g kg^−1^), MD (2.88–3.89 g kg^−1^), and HD (1.99–2.65 g kg^−1^) plots (*P* < 0.05; Fig. 2A). Additionally, sampling time had a significant impact on TN content, with higher levels observed in August compared to May and June across the 0–20 cm soil layer (except for the 0–10 cm layer at the ND plot). However, there was no significant difference in TN content among the various levels of degradation in June.

The variations in NH_4_^+^-N content were consistent across soil layers at different levels of vegetation degradation (Fig. 2B). The NH_4_^+^-N content was significantly reduced in the MD and HD plots compared to the ND and SD plots (*P* < 0.05). Specifically, the NH_4_^+^-N content in the 0–10 cm soil layer exhibited the following pattern: ND (3.67–5.54 g kg^−1^) > SD (2.58–3.87 g kg^−1^) > MD (1.97–3.49 g kg^−1^) > HD (0.48–1.38 g kg^−1^). In the 10–20 cm layer, the NH_4_^+^-N content was as follows: ND (2.01–4.72 g kg^−1^) > SD (1.72–3.81 g kg^−1^) > HD (0.62–2.58 g kg^−1^) > MD (0.42–1.59 g kg^−1^). Soil NH_4_^+^-N content showed significant variations over the study period. The NH_4_^+^-N content was significantly higher in August compared to May and June (*P* < 0.05), except for the HD plot.

During vegetation degradation, the NO_3_^−^-N content exhibited an increasing trend in both August and May (Fig. 2C), while it decreased in June. Compared to the ND and SD plots, the NO_3_^−^-N content was significantly higher in the MD and HD plots (*P* < 0.05). In June, the NO_3_^−^-N content reached 8.79 g kg^−1^ in the 0–10 cm soil layer at the SD plot and 8.41 g kg^−1^ in the 10–20 cm layer, which was significantly higher than that in the ND, MD, and HD plots. The sampling time significantly influenced NO_3_^−^-N across different levels of degradation, with the highest concentrations occurring in June for ND, SD, and MD samples and in August for MD samples.

### Spatial and temporal differences in the abundance of NFGs during vegetation degradation

**Fig 3:**
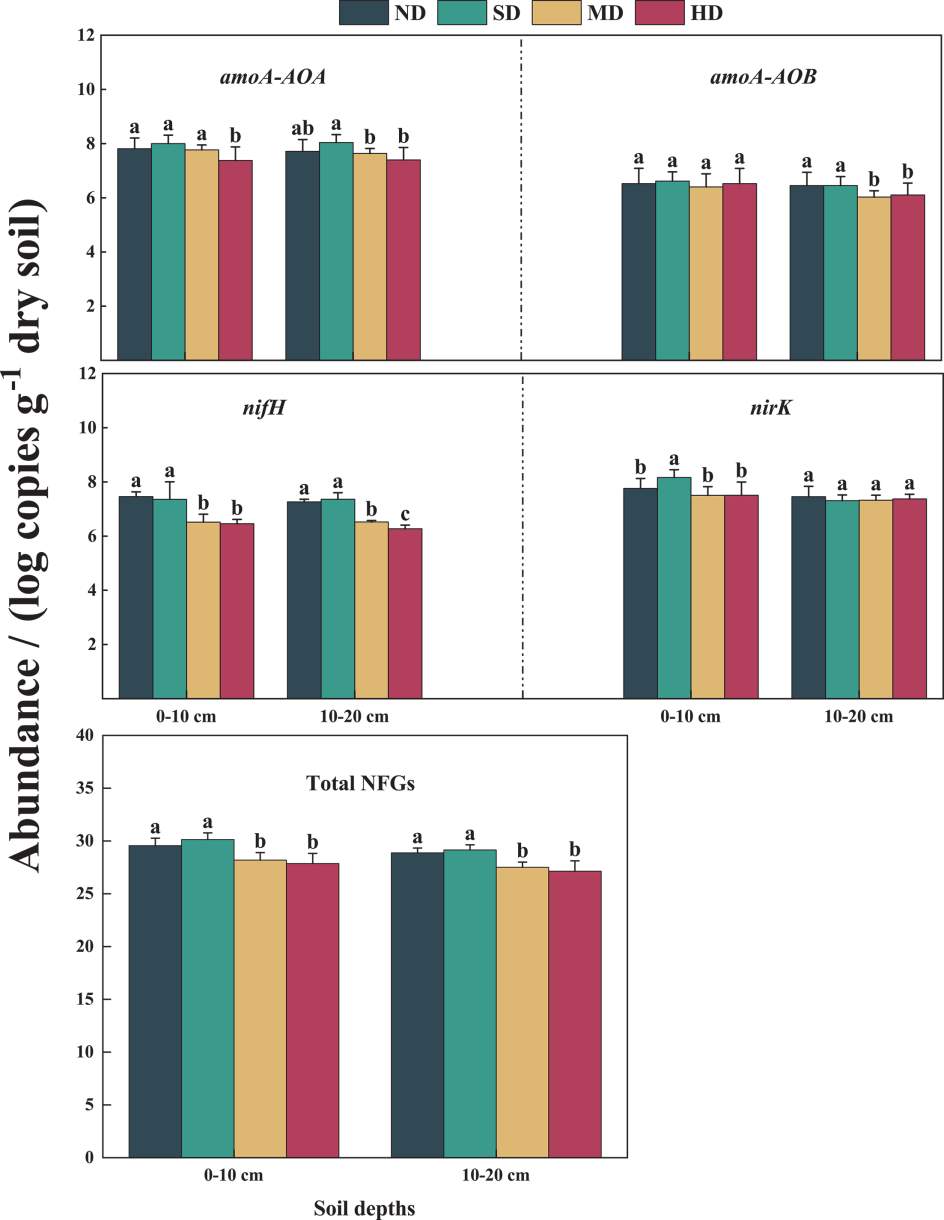
Abundance of NFGs under different levels of vegetation degradation in 2020 and 2021. Different lowercase letters indicate that under the same soil layer, there are significant differences among different vegetation degradation samples (*P* < 0.05).

During vegetation degradation, variations in the average abundance of NFGs were observed (Fig. 3). Notably, the average abundance of *amoA-AOA*, *nifH*, and TNFGs was significantly lower in the MD and HD plots compared to the ND and SD plots. Conversely, the average abundance of *amoA-AOB* at the 0–10 cm soil layer and *nirK* at the 10–20 cm soil layer did not show significant differences across the different levels of degradation (Fig. 3). In terms of gene copy numbers, the *amoA-AOA* gene had a copy number ranging from 7.38 to 7.81 log_10_ per gram dry soil, the *nirK* gene had a copy number ranging from 7.30 to 8.16 log_10_ per gram dry soil, and the *amoA-AOB* gene had a copy number ranging from 6.27 to 6.52 log_10_ per gram dry soil.

**Fig 4:**
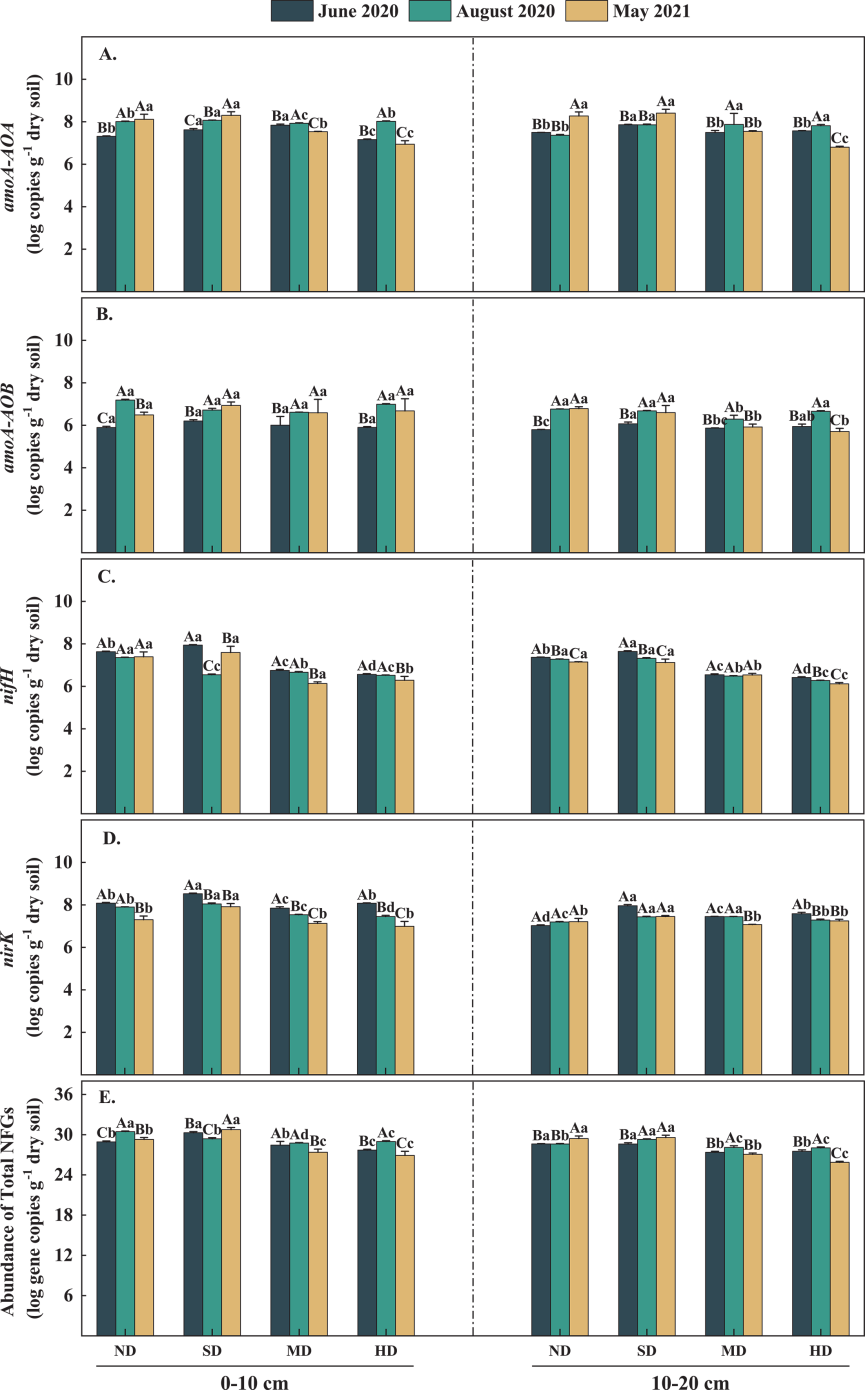
Changes of functional gene abundance of soil nitrogen transformation under different vegetation degradation levels during different observation periods in 2020 and 2021. Different lowercase letters indicate that there are significant differences between different points at the same time, different uppercase letters indicate that there are significant differences between the same points at different times, the significance is *P* < 0.05, and the vertical bar is SE (*n* = 3).

Significant variations in the abundance of NFGs were observed across different sampling times and levels of vegetation degradation (*P* < 0.05, Table 2). In the 0–10 cm soil layer, the copy number of *amoA-AOA* genes ranged from 6.94 to 8.30 log_10_ per gram dry soil, the copy number of *nifH* genes ranged from 6.13 to 7.93 log_10_ per gram dry soil, and the TNFGs ranged from 26.90 to 30.74 log_10_ per gram dry soil (Fig. 4A, C and E). Notably, the abundance of *amoA-AOA*, *nifH*, and TNFGs was significantly lower in the HD and MD plots compared to the ND plot (*P* < 0.05). However, no significant differences were observed between ND and SD plots. Except for the performance of TNFGs abundance in the SD plot, the *amoA-AOA* and TNFGs exhibited a higher gene copy number in August 2020. The *nifH* gene copy number was higher in June 2020. The *amoA-AOB* gene copy number ranged from 5.89 to 6.93 log_10_ per gram of dry soil during vegetation degradation, showing fluctuations; however, in the 0–10 cm soil layer, vegetation degradation did not significantly impact the *amoA-AOB* gene copy number (*P* > 0.05; Fig. 4B). The gene copy number of the *nirK* gene in the SD plot ranged from 7.91 to 8.52 log_10_ per gram dry soil, which was significantly higher than in the ND plot (7.30­8.08 log_10_ per gram dry soil), the MD plot (7.12­7.84 log_10_ per gram dry soil), and HD plot (6.99­8.08 log_10_ per gram dry soil; Fig. 4D). In sampling time, the *nirK* gene copy number exhibited a pattern in June 2020, August 2020, and May 2021, in contrast to the pattern seen with the *amoA-AOB* gene copy number.

**TABLE 2:** Results of the Generalized Linear Model (GLM)-repeated measures for the abundance differences in the NFGs among vegetation degradation levels using time as the repeated variable

Vegetation degradation level		*amoA-AOA*		*amoA-AOB*		*nifH*		*nirK*		Total NFGs	
		F	Sig.	F	Sig.	F	Sig.	F	Sig.	F	Sig.
VL^*a*^	0–10 cm	40.03	0.000	0.71	0.572	163.35	0.000	51.98	0.000	65.28	0.000
ST^*b*^		109.12	0.000	35.86	0.009	44.58	0.000	266.08	0.000	28.52	0.000
VL × ST		53.52	0.000	2.16	0.103	29.20	0.000	8.20	0.006	28.82	0.000
VL	10–20 cm	73.26	0.000	22.81	0.000	839.88	0.000	5.27	0.027	116.46	0.000
ST		11.16	0.006	82.21	0.000	56.73	0.000	113.22	0.000	35.01	0.000
VL × ST		92.39	0.000	15.90	0.000	9.43	0.005	140.740	0.000	59.45	0.000

^*a*^ VL, vegetation degradation level.

^*b*^ ST, sampling time.

During the sampling times, the variation in *amoA-AOA*, *nifH*, and TNFGs genes in the 10–20 cm soil layer was similar to the trends observed in the 0–10 cm layer across different levels of vegetation degradation (Fig. 4A, C and E). The abundance of *amoA-AOA* (7.36–8.27 log_10_ per gram of dry soil), *nifH* (7.14–7.37 log_10_ per gram of dry soil), and TNFGs (28.59–29.41 log_10_ per gram of dry soil) in the 10–20 cm layer at the ND plot was significantly higher than those observed at the HD and MD plots. Specifically, *amoA-AOA* gene copy numbers ranged from 7.49 to 7.87 log_10_ per gram of dry soil in ND plot and 6.80 to 7.81 log_10_ per gram of dry soil in MD plot, *nifH* gene copy numbers were between 6.53 and 6.54 log_10_ per gram of dry soil in HD plot and 6.27 to 6.41 log_10_ per gram of dry soil in MD plots, and TNFGs ranged from 27.07 to 28.09 log_10_ per gram of dry soil in HD plots and 25.87 to 28.03 log_10_ per gram of dry soil in MD plots (Fig. 4A, C and E). However, *amoA-AOA*, *nifH*, and TNFGs gene copy numbers were not significantly different between SD and HD plots. Higher levels of *amoA-AOA* and TNFGs gene copy numbers were observed in August 2020 and May 2021. Conversely, higher *nifH* gene copy numbers were recorded in June 2020. In the 10–20 cm soil layer, the copy number of the *amoA-AOB* gene was significantly lower in the MD (5.87–6.29 log_10_ per gram dry soil) and HD (5.70–6.66 log_10_ per gram dry soil) plots compared to the ND plot (5.79–6.78 log_10_ per gram dry soil; *P* < 0.05). However, there was no significant difference when compared to the SD plot (6.06–6.69 log_10_ per gram dry soil; Fig. 4B). The *nirK* gene copy number in the SD plot (7.43–7.96 log_10_ per gram dry soil) was significantly higher than in the ND (7.02–7.20 log_10_ per gram dry soil), MD (7.07–7.45 log_10_ per gram dry soil), and HD (7.24–7.58 log_10_ per gram dry soil) plots (*P* < 0.05; Fig. 4D). In terms of temporal variation, a higher *nirK* gene copy number was observed only in June and August of 2020 in the MD and HD plots.

Except for the *amoA-AOB* genes in the 0–10 cm layer, the repeated-measures ANOVA indicated significant interaction effects between sampling time and vegetation degradation level on all NFGs (*P* < 0.05). Additionally, the analysis revealed that sampling time was significantly associated with variations in soil nitrogen-related functional genes (Table 2).

### The relative abundances of NFGs along the vegetation degradation level

**Fig 5:**
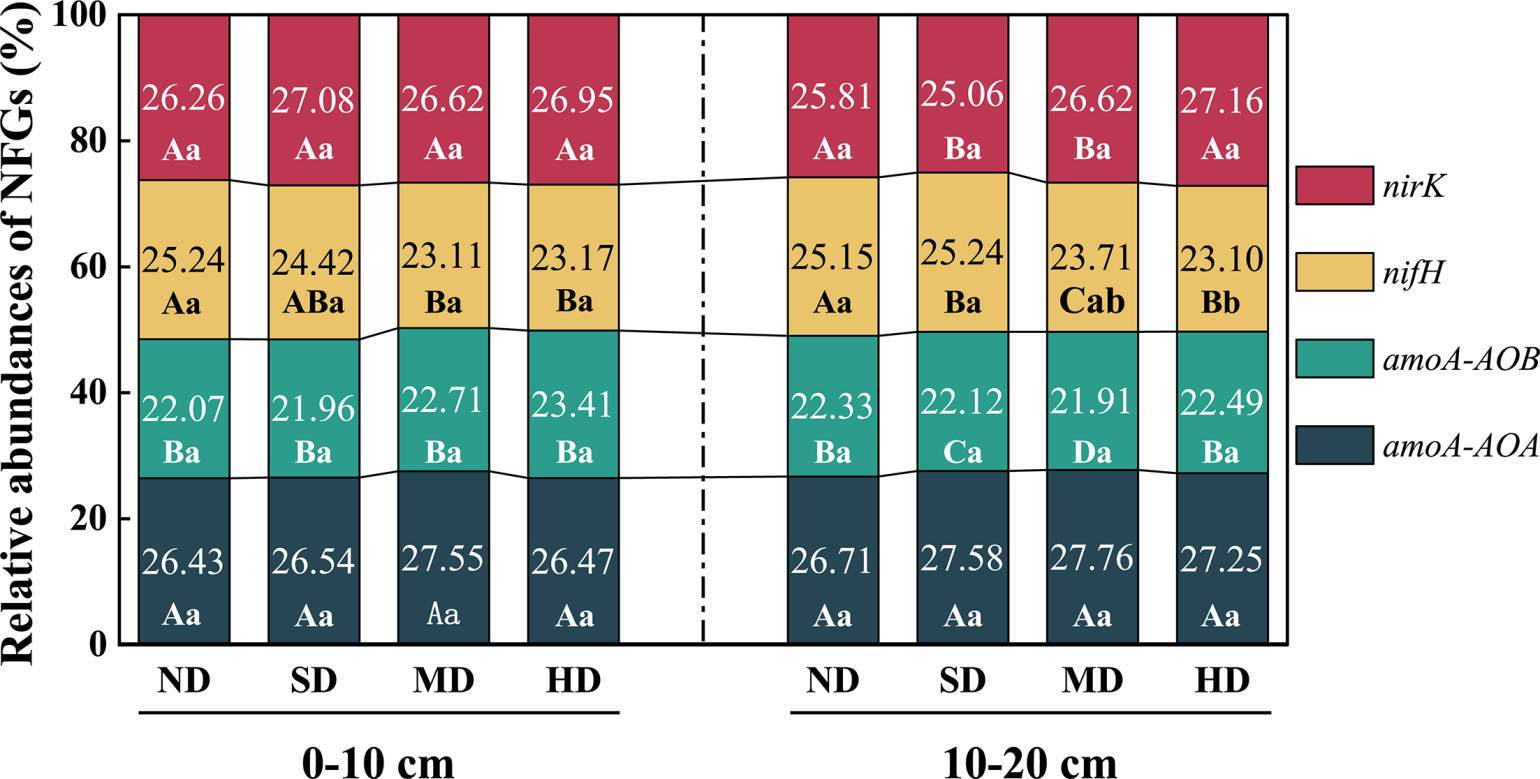
The relative abundance of soil nitrogen transformation functional genes along vegetation degradation levels. Different lowercase letters indicate significant differences (*P* < 0.05) between different sites for the same gene. Different uppercase letters indicate significant differences between the same genes under the same treatment (*P* < 0.05).

Figure 5 shows the distribution of relative abundance of NFGs under different levels of vegetation degradation. Within the 0–10 cm and 10–20 cm soil layers, the relative gene abundances of *amoA-AOA* and *nirK* ranged from 52.52% to 54.41%, significantly higher than those of *amoA-AOB* and *nifH*, which ranged from 45.59% to 47.48% (*P* < 0.05). Notably, *amoA-AOB* abundances were consistently below 23%. Specifically, in the 0–10 cm soil layer, no significant differences were observed in the relative gene abundances of *amoA-AOA*, *amoA-AOB*, *nifH*, and *nirK* across degradation gradients (*P* > 0.05). However, excluding *nifH*, a trend of increasing relative gene abundances for *amoA-AOA*, *amoA-AOB*, and *nirK* was noted with increasing degradation. In the 10–20 cm soil layer, the abundance of *nifH* was significantly higher in HD plots compared to ND and SD plots (*P* < 0.05). The absence of significant differences in the abundances of other genes across degradation levels indicates a selective impact of degradation on specific N cycling functions. The trends were similar to those in the 0–10 cm layer. Compared to ND plots, degraded sites exhibited increases in the relative proportions of *amoA-AOA*, *amoA-AOB*, and *nirK* by 0.2%–4.2%, 0.72%–6%, and 1.37%–5.23%, respectively. Conversely, the relative proportion of *nifH* decreased by 6.07%–9.22%. As a result, the communities of ammonia-oxidizing microbes (*amoA-AOA*, + *amoA-*AOB) and the *nirK* community showed enhanced nitrification and denitrification functions in the degraded sample plots, while N fixation functions gradually declined.

### Relationships between the absolute abundance of the NFGs and the environmental factors

**Fig 6:**
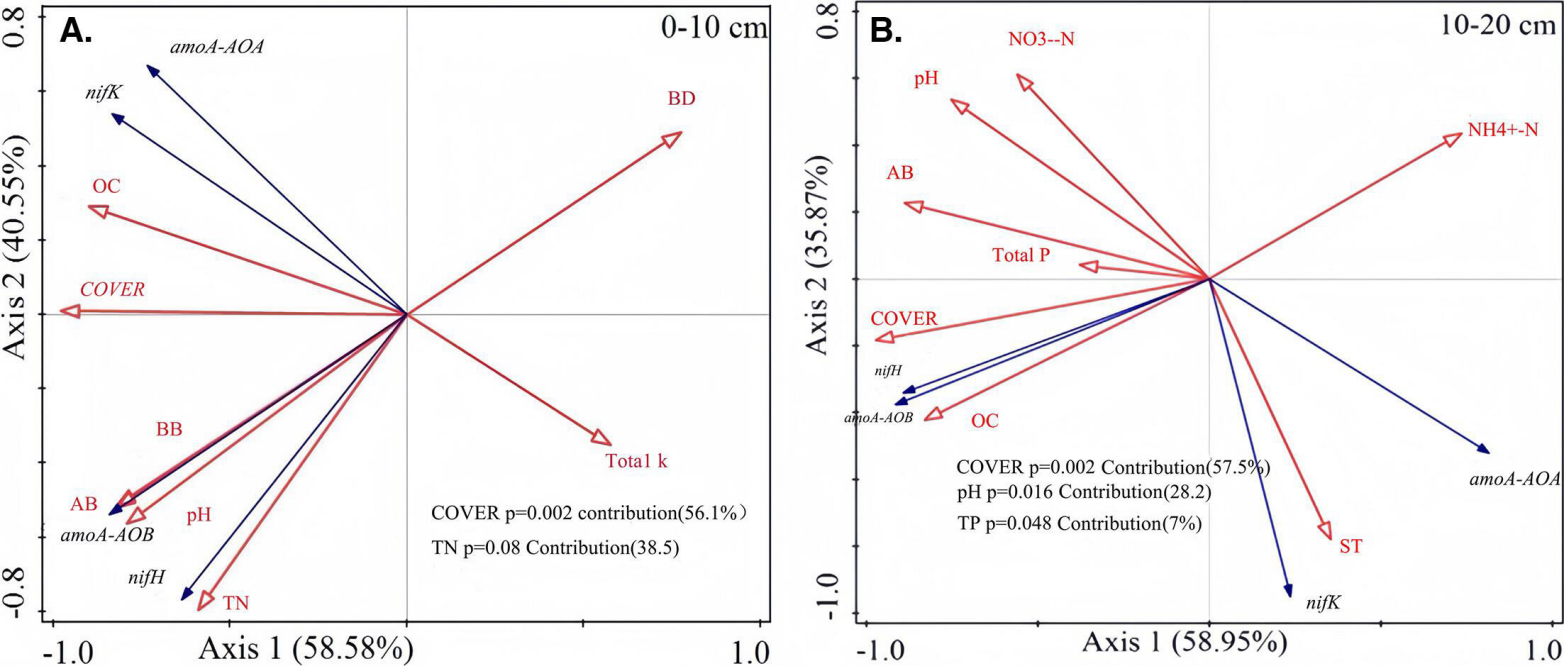
Redundancy analysis of the relationship between the absolute abundance of NFGs and environmental factors. OC, organic carbon; COVER, vegetation coverage; ST, sample time.

The RDA ordination diagram further illustrates that the variations in NFGs in both the 0–10 cm and 10–20 cm soil layers were predominantly influenced by the combined effects of vegetation degradation. The data indicate that vegetation growth, along with changes in soil physical and chemical properties, accounted for a substantial part of the variation, contributing 99.13% in the 0–10 cm layer and 94.82% in the 10–20 cm layer (Fig. 6A and B). In the 0–10 cm soil layer, the abundance of NFGs was primarily associated with plant characteristics such as vegetation cover, AB, and BB, as well as soil traits including SOC, pH, TN, TK, and BD. In the 10–20 cm soil layer, the abundance of NFGs was regulated by vegetation characteristics, including vegetation cover and AB, as well as soil nutrients such as SOC, TP, and NH_4_^+^-N, NO_3_^−^-N. Additionally, soil physical properties like pH and soil temperature also played significant roles in influencing NFG abundance. Vegetation cover, TN, TP, and pH were significant regulators of the abundance of NFGs. Specifically, vegetation cover and TN were the most substantial contributors, accounting for over 94% of the variation in the 0–10 cm soil layer. In the 10–20 cm soil layer, vegetation cover, TP, and pH collectively explained 92% of the variation in NFGs.

## DISCUSSION

### Vegetation degradation changes the relationship between NFGs and N components

Our results indicate that TN content decreases progressively with increasing vegetation degradation, supporting the notion that the levels of vegetation degradation impede plant production (33). The decline in plant biomass, as documented in Table 1, negatively impacts soil structure and hampers nutrient cycling within the soil (34, 35). This disruption in soil dynamics subsequently results in reduced TN content (25). Our study found a significant correlation between the *nifH* gene and TN content (Fig. 6), indicating that the potential capacity of N-fixing microorganisms decreases with more intense vegetation degradation (36). This observation aligns with findings from Song et al. (2019a), who reported that reduced expression of *nifH* genes leads to a diminished capacity for symbiotic N fixation, ultimately resulting in lower TN content (11). However, these findings contrast with those of Bu et al. (2020a), who reported an increase in the N fixation potential of *nifH* genes during soil desertification (7). This discrepancy could be attributed to environmental differences between the study areas. This could be because the soil plots that we collected may not adequately represent the inter-vegetative rhizosphere soil. Although we detected free N-fixing bacteria in the soil (37), these bacteria still play a significant role in N turnover during vegetative growth (10, 37).

With intensified vegetation degradation, NO_3_^−^-N content gradually increased, showing an opposite trend to NH_4_^+^-N content (Fig. 2B and C). NO_3_^−^-N content increases due to reduced nutrient inputs and reduced above- and belowground vegetation biomass as vegetation is degraded. Reduced vegetative biomass, which impacts the incorporation of organic matter into the soil, is likely to significantly affect the process of soil organic N mineralization. This alteration can hinder or slow down N transformations, thereby changing the dynamic balance between different forms of N in the soil (38). Additionally, as the levels of vegetation degradation increases, the soil water content tends to decrease. This reduction in moisture creates conditions favorable for the aerobic activity of soil-nitrifying microorganisms, facilitating the accumulation of NO_3_^−^-N in the degraded plots (39). This dynamic illustrates how changes in soil water content and oxygen levels critically influence N cycling, impacting the conversion rates and forms of N present in the ecosystem.

From the perspective of N-cycling microorganisms, the composition of N-cycling microbial gene communities provides insights into the dynamics of N turnover processes (40, 41). Ammonia-oxidizing microorganisms, specifically *amoA-AOA* (archaea) and *amoA-AOB* (bacteria), are important in indicating nitrification processes, which convert ammonia to nitrates (42). A higher proportion of these microbial communities enhances the conversion of NH_4_^+^-N to NO_3_^−^-N, facilitating the nitrification process (7). In our study, the relative gene abundance of ammonia-oxidizing microbial communities (*amoA-AOA* and *amoA-AOB*) was higher in the MD and HD plots compared to the ND plot (Fig. 5). Additionally, alongside the observed increase in nitrification markers, there was also a slight increase in the relative abundance of *nirK* genes, which are associated with the denitrification process, indicating a complex interaction between nitrification and denitrification processes under varying environmental conditions. As a result, the process of nitrate reduction to N gases (such as N_2_, N_2_O, and NO) and their escape from the soil was enhanced (7), which occurred alongside increased accumulation of NO_3_^−^-N.

Seasonal variations significantly affect N compound concentrations. Higher concentrations of N compounds were observed in August 2020 compared to June 2020 and May 2021, primarily due to the enhanced microbial activity associated with higher soil temperatures. These temperatures promote the decomposition of vegetative litter, which in turn increases TN and NH_4_^+^-N content (43). However, the change in NO_3_^−^-N content did not follow this response pattern. At the end of the vegetation growth period, plant senescence and the decomposition of apoplastic material result in a more abundant substrate being available to soil microorganisms, thereby enhancing the accumulation of soil nutrients (44–46). During winter, lower temperatures create freezing conditions that stabilize the environment for microorganisms. This potentially provides conditions that support the maintenance of microbial activity despite the cold (46, 47). Following the soil thawing process in the coming year, significant leaching of organic matter occurs, enhancing the effectiveness of soil organic matter (48–50). This process provides ample resources for soil microorganisms, particularly ammonia-oxidizing microbial communities. As a result, there is likely to be an increase in the copy number of *amoA-AOA* and *amoA-AOB* genes, reflecting heightened microbial activity related to ammonia oxidation (51). As soil temperature and water content rise, conditions become more favorable for microbial life (52). In June 2020, the *nifH* gene was particularly active (Fig. 4C), enhancing soil N fixation. Enhanced soil N fixation supplies available energy to the flora involved in the ammonia oxidation process, thereby facilitating the conversion of NH_4_^+^-N to NO_3_^−^-N. This process is crucial for the N cycle, enabling the efficient utilization of N by plants (53). Concurrently, during the warming period of June, the activity of the *nirK* gene related to denitrification became more pronounced, stimulating the soil N cycle and enhancing the functions of *amoA-AOA* and *amoA-AOB*. This series of interactions resulted in a net increase in NO_3_^−^-N content relative to NH_4_^+^-N.

In our study, the response of the *amoA-AOB* gene to environmental changes was more pronounced (Fig. 5), likely due to its limited phylogenetic diversity and high sensitivity to environmental fluctuations (54–56). Existing studies have shown that the level of soil nitrate content is positively correlated with the nitrification activity of the *amoA-AOB* gene community, indicating that higher activity within this community often leads to increased nitrate concentrations in the soil (57). This interrelationship underscores the crucial role of the *amoA-AOB* gene community in the nitrification process, which is instrumental in regulating the overall level of soil nitrate. This community facilitates the conversion of ammonium to nitrate, directly influencing soil nitrate concentrations. Inhibition of *amoA-AOA* gene activity occurs at higher concentrations of NH_4_^+^-N (58). This suggests that *amoA-AOB* genes dominate the nitrification process during vegetation degradation.

### Effect of vegetation degradation on NFGs

Our study demonstrated that NFGs declined to varying levels with increasing vegetation degradation (Fig. 3). This decline is closely related to changes in both aboveground biomass and soil nutrient levels, as confirmed by findings from Song et al. (2019b), who reported that these changes highlight the interconnectedness of vegetation health and microbial functions involved in soil N cycling (11). This interconnectedness is further illustrated by the community sensitivity of ammonia-oxidizing microorganisms, which respond differently to environmental changes during vegetation degradation. Compared to other N-cycle gene groups, the *amoA-AOA* genes have a higher metabolic capacity and greater adaptability, enabling them to respond more effectively to low-soil substrates (53, 59). Interestingly, *amoA-AOA* may not rely solely on NH_4_^+^ as its energy source, as suggested by Di et al. (60), and might obtain energy from alternative sources (61), such as soil organic matter, thereby reducing its direct dependence on soil N (26). Further supporting these variations in microbial adaptability, a quantitative meta-analysis indicated that *amoA-AOB* abundance was significantly reduced by 31.0% in forest and grassland ecosystems rich in vegetative species (26). These findings underscore the differing adaptive properties of ammonia-oxidizing microbial communities to various habitats, resulting in lower abundances of *amoA-AOA* and *amoA-AOB* in better-vegetated habitats, such as those in the ND and SD plots. Despite these variations, the differences in gene abundances were not significantly different, highlighting the overarching influence of environmental conditions on microbial community dynamics.

*nifH*, which is crucial for fixing atmospheric N into a usable form, requires substantial amounts of energy to facilitate N access. Similarly, the *nirK* gene, which is involved in the process of denitrification, also demands significant energy inputs (7, 62, 63). These energy requirements are often met by the availability of soil C resources, which serve as a vital energy source for these microbial processes. Our study identified significant correlations between the abundance of *nifH* and *nirK* genes and SOC, as well as with above- and belowground biomass (Fig. 6). This is because species diversity enhances ecological niche complementarity and increases aboveground primary productivity, thereby maintaining soil N turnover (64, 65). However, this is a dynamic equilibrium process, and excess N can inhibit the accumulation of *nifH* (26). The *nifH* gene copy number remained relatively stable in the ND and SD plots, showing significant differences when compared to the MD and HD plots (Fig. 3). This stability suggests that *nifH* is less affected by minor disturbances in well-maintained ecosystems. Additionally, the *nirK* gene appears to adapt its survival strategy according to the level of vegetation degradation, as indicated by its varying abundances across different degradation levels (8, 66). This adaptability underlines the gene critical role in responding to environmental changes and stresses. Soil surfaces rich in SOC are more favorable for *nirK* accumulation in the habitat selection of *nirK* bacteria (67, 68). In addition, the concentration of substrates necessary for the *nirK*-mediated reactions is diminished due to uptake, utilization, leaching, and emission of NO and N_2_O by plants. This reduction in substrate availability can dilute the *nirK* gene copy number in N-rich soils, impacting the gene’s presence and activity levels to some extent (69, 70). This trend was more pronounced in the SD plots, suggesting that vegetation degradation may enhance the denitrification potential of the soil. Microbial communities maintained a strong response to short-term adverse environmental changes.

### The intrinsic driving force of NFGs abundance distribution with soil depth

The vertical distribution characteristics of microorganisms in soil are closely associated with differences in soil properties between layers (71). It has been observed that microbial activity is typically lower in deeper soil layers, primarily due to the limited availability of C and N sources (72, 73). In our study, the abundance of NFGs in the 0–10 cm soil layer was significantly influenced by vegetation cover and TN content, which contributed to 56.1% and 38.5% of the variations in soil physicochemical properties, respectively. In the 10–20 cm layer, vegetation cover accounted for 57% of the regulation, pH for 28.2%, and TP content for 7% of the variations (Fig. 6).

In the 0–10 cm soil layer, an increase in soil water content, soil temperature, and vegetation cover promotes an increase in soil C and N content. This enhancement in soil nutrients supports overall soil fertility, stimulates microbial activity, and facilitates nutrient cycling (25). These topsoil conditions are usually favorable for microbial survival and provide an optimal environment for NFGs to survive. However, these factors do not account for the observed lower *nifH* gene copy number in August 2020 in the 0–10 cm soil layer. The *nifH* gene, which is involved in N fixation, is sensitive to phosphorus availability (74). In the SD plot, the TP content was at a moderate level, which may not have been sufficient to support optimal N fixation. This could explain why the *nifH* gene copy number in the SD plot was similar to that observed in the HD plot. In addition, NH_4_^+^-N content is often regarded as a limiting factor for *nifH* abundance. Higher NH_4_^+^-N content causes *nifH* genes to shut down the N-fixation pathway in order to avoid energy waste (75, 76). Lower NH_4_^+^-N levels in degraded plots contributed to the increase in *nifH* activity. However, the higher *nifH* gene copy number in the ND plot is due to the soil organic carbon replenishment will still provide *nifH* microbial viability (62, 63).

In the 10–20 cm soil layer, the relative abundance of the *amoA-AOA* gene remained at a higher level (Fig. 5). Similarly, the abundance of the *nirK* gene is influenced by soil environmental conditions, akin to *amoA-AOA*. Both the *nirK* and *amoA-AOA* genes are capable of deriving energy from soil C sources, which support their metabolic activities and contribute to their roles in N-cycling nitrification by *amoA-AOA* and denitrification by *nirK* (67, 68). Notably, in the 10–20 cm soil layer, the relative gene abundance of *amoA-AOA* increased, demonstrating enhanced nitrification activity at this depth. Meanwhile, the relative gene abundance of *nirK* increased specifically in the MD and HD plots (Fig. 5). This is due to the ability of the *nirK* gene community to select ecological niches under different habitat conditions (66). This also implies that the nitrification and denitrification potential of the soils in the MD and HD plots increased to some extent with vegetation degradation.

Soil pH is a critical factor influencing microbial diversity and abundance (77, 78). Generally, lower soil pH is associated with a reduced abundance of *nirK* and *nifH* genes, reflecting the pH sensitivity of these microbial communities. While our results support this trend for the *nirK* gene, the *nifH* gene did not follow the expected pattern. This discrepancy could be attributed to several factors, including variations in soil nutrient levels and broader environmental changes that might differentially affect the microbes responsible for N fixation compared to those involved in denitrification (8, 53, 66). When there is insufficient N and phosphorus in the 10–20 cm soil layer, *nifH* microbes activate alternative resource collection strategies to maintain N-fixation capacity (37, 79).

In summary, the abundance of NFGs varied in response to the process of wetland vegetation degradation and soil depth, predominantly influenced by vegetation biomass, C, and N content. These variations reflect the adaptation of N cycle-related gene communities to differing environmental conditions and the interactions between ecological niches during vegetation degradation. This highlights how microbial communities adapt to changes in their habitat, with potential implications for N-cycling processes in degraded wetland ecosystems (7, 80).

### Conclusions

Our study shows that vegetation degradation in alpine wet meadows not only reduces the content of soil N components but also alters the cycling of soil nutrients. This leads to a decrease in the abundance of functional genes related to the N cycle, with notable differences in response to spatial and temporal variations. We observed that changes in gene abundance were distinct from shifts in soil physicochemical properties. Plant cover significantly influenced the abundance of NFGs within the 0–20 cm soil layer, while TN content, pH value, and TP content all played roles in regulating NFGs abundance. Even with intensified vegetation degradation, the degraded plots maintained the potential for nitrification and denitrification. Overall, our results elucidate how the process of vegetation degradation in alpine wet meadows impacts NFGs and highlights the interactions of these genes under varying levels of vegetation degradation.

## SUPPLEMENTAL MATERIAL

Figure S1 aem.00813-24-s0001.tif Distribution diagram of the actual study area and sample sites in northeastern Tibetan Plateau. A, B, and C represent the three transects extending outward from Gahai Lake. ND, non-degraded; SD, slightly degraded; MD, moderately degraded; HD, heavily degraded.

Supplemental tables aem.00813-24-s0002.docx Tables S1 to S3.

ASM does not own the copyrights to Supplemental Material that may be linked to, or accessed through, an article. The authors have granted ASM a non-exclusive, world-wide license to publish the Supplemental Material files. Please contact the corresponding author directly for reuse.
