# Supplementary figures and images for: Soil nitrogen-related functional genes undergo abundance changes during vegetation degradation in a Qinghai-Tibet Plateau wet meadow
Source: Appl Environ Microbiol. 2024 Sep 20;90(10):e00813-24. doi: 10.1128/aem.00813-24 (PMC11497797; doi:10.1128/aem.00813-24)

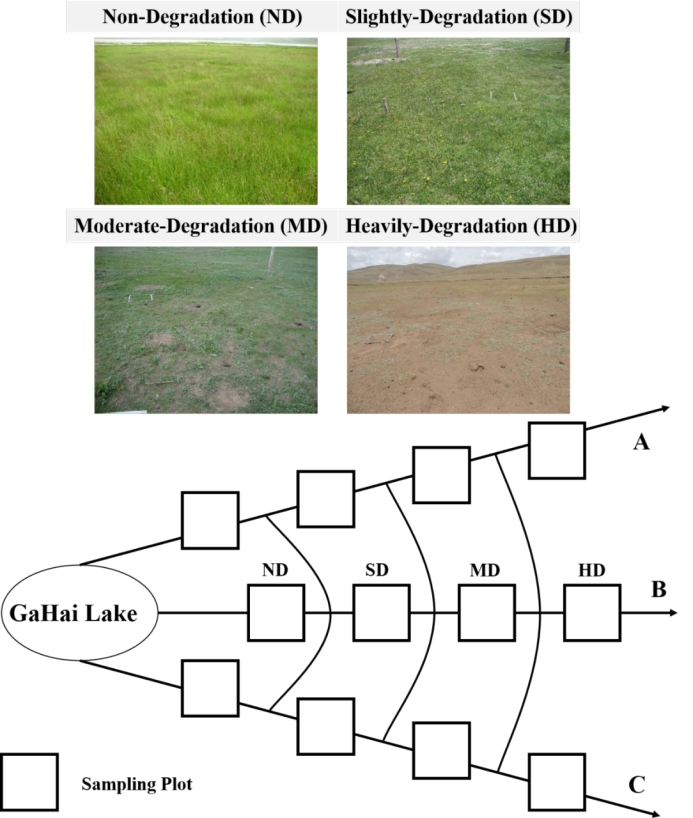

Supplement: Figure S1 — Distribution diagram of the actual study area and sample sites in northeastern Tibetan Plateau. A, B, and C represent the three transects extending outward from Gahai Lake. ND, non-degraded; SD, slightly degraded; MD, moderately degraded; HD, heavily degraded. [file aem.00813-24-s0001.tif]
