# Supplementary material for: Soil nitrogen-related functional genes undergo abundance changes during vegetation degradation in a Qinghai-Tibet Plateau wet meadow
Source: Appl Environ Microbiol. 2024 Sep 20;90(10):e00813-24. doi: 10.1128/aem.00813-24 (PMC11497797; doi:10.1128/aem.00813-24)
Supplement: Supplemental tables — Tables S1 to S3. [file aem.00813-24-s0002.docx]

| **Table S1**  **Description of the experimental sites.** | | | | | |
| --- | --- | --- | --- | --- | --- |
| Vegetation degradation level | Altitude (m) | Distance from the lake（m） | Cover (%) | groundwater level (cm) | Dominant species |
| ND | 3477 | 30-50 | >90 | 20-40 | *Kobresia tibetica*+*Potentilla anserine+ Poa annua* |
| SD | 3478 | 70-80 | 75-90 | 40-70 | *Carex* sp*.*+*Potentilla anserine+Artemisia frigida Willd* *+Kobresia capilifolia* |
| MD | 3484 | 100-120 | 30-70 | >70 | *Artemisia frigida Willd* +*Artemisia sacrorum*.var. + *Kobresia capilifolia* |
| HD | 3486 | 130-150 | Low groundwater level, overgrazing, and severe rodent damage resulted in sparse vegetation, with only little *Artemisia frigida* Willd. And *Polygonum viviparum* | | |
| ND, non-degraded; SD, slightly-degraded; MD, moderately-degraded; HD, heavily-degraded. Abbreviations also apply to the table below | | | | | |

| **Table S2**  Changes in soil physicochemical properties in the soil profile (0-10 cm, 10-20 cm) during the process of degraded vegetation. Different lower case letters indicate significant differences (*P*<0.05) between the same soil horizons for the same indicator measured. | | | | | |
| --- | --- | --- | --- | --- | --- |
| Parameters | Depth  (cm) | ND | SD | MD | HD |
| Organic C  (g kg^-1^) | 0-10 | 65.23±2.87a | 66.06±2.72a | 30.63±2.57b | 25.95±1.90b |
|  | 10-20 | 42.25±3.09a | 42.61±2.30a | 28.94±5.34b | 31.65±4.58b |
| Total P  (g kg^-1^) | 0-10 | 3.02±0.06a | 2.72±0.25ab | 2.52±0.23b | 2.51±0.14b |
|  | 10-20 | 2.97±0.12a | 2.64±0.18a | 2.75±0.34a | 1.05±0.06b |
| Total K  (g kg^-1^) | 0-10 | 5.83±0.53a | 5.66±0.01a | 5.66±0.02a | 6.40±0.39a |
|  | 10-20 | 5.64±0.02b | 5.87±0.39ab | 6.28±0.34a | 5.71±0.20b |
| pH | 0-10 | 7.94±0.07a | 7.69±0.02b | 7.65±0.02b | 7.67±0.01b |
|  | 10-20 | 7.95±0.03a | 7.73±0.01b | 7.74±0.01b | 7.75±0.01b |
| Bulk density  (g m^-3^) | 0-10 | 0.73±0.01c | 0.89±0.04b | 0.93±0.05b | 1.05±0.06a |
|  | 10-20 | 0.82±0.03b | 0.99±0.04a | 1.06±0.06a | 1.04±0.06a |

**Table S3**

Primers for PCR amplification

| Name | Sequence | Types | PCR  Products  (bp) |
| --- | --- | --- | --- |
| *amoA*-AOA | STAATGGTCTGGCTTAGACG | Forward primer | 635 |
|  | GCGGCCATCCATCTGTATGT | Reverse primer |  |
| *amoA*-AOB | GGGGTTTCTACTGGTGGT | Forward primer | 491 |
|  | CCCCTCGGGAAAGCCTTCTT | Reverse primer |  |
| *nifH* | AAAGGCGGAATCGGCAAGTC | Forward primer | 458 |
|  | TTGTTCCGCGGCGTACATG | Reverse primer |  |
| *nirK* | GGMATGGTKCCSTGGCA | Forward primer | 515 |
|  | GCCTCGATCAGRTTRTGG | Reverse primer |  |
